# Knowledge, Awareness, and Attitude Regarding Obstructive Sleep Apnea Among Primary Healthcare Physicians in the Al-Baha Region of Saudi Arabia

**DOI:** 10.7759/cureus.51435

**Published:** 2024-01-01

**Authors:** Mohammed A. Alghamdi, Rajab Alzahrani, Mujtaba A. Ali, Ziyad Alharthi, Ahmed M Alyahya, Abduljawad H Alghamdi, Basil N Alghamdi, Wejdan Alghamdi

**Affiliations:** 1 Unit of Otolaryngology, Department of Surgery, Faculty of Medicine, Al-Baha University, Al-Baha, SAU; 2 Department of Medicine, Al-Baha University, Al-Baha, SAU; 3 Department of Medicine, Al-Baha University, Al Baha, SAU; 4 Family Medicine, King Faisal Specialist Hospital and Research Centre, Jeddah, SAU

**Keywords:** healthcare workers awareness, healthcare workers (hcw), primary healthcare physicians, nocturnal snoring, attitudes and practices, obstructive sleep apnea (osa)

## Abstract

Introduction: Obstructive sleep apnea (OSA) is a clinically significant disorder that requires attention from healthcare professionals. This study aimed to assess the knowledge, awareness, and attitude of primary healthcare physicians regarding OSA in Saudi Arabia.

Methodology: A cross-sectional observational study design was employed from January 18, 2023, to August 18, 2023, focusing on primary healthcare physicians practicing in the Al-Baha region of Saudi Arabia. The survey included questions related to knowledge, awareness, and attitudes toward OSA, using the Obstructive Sleep Apnea Knowledge and Attitude (OSAKA) questionnaire for OSA, and demographic information. Descriptive statistics and chi-square tests were used to analyze the data.

Results: A total sample size of 174 primary health care physicians was collected, where the majority of participants were male (101, 58.0%), and the mean age of the participants was 32.73 years (SD = 9.14). The item with the most correct answers was "The majority of patients with obstructive sleep apnea snore" (150, 86.2%). On the other hand, the item with the lowest number of correct answers was "Laser-assisted uvulopalatoplasty is an appropriate treatment for severe obstructive sleep apnea" (17, 9.8%). The majority of participants agreed that it is essential for physicians to know about OSA as a clinical disorder (161, 92.5%). However, when it came to screening all patients for possible OSA, there was a higher proportion of participants who disagreed or strongly disagreed (111, 63.8%). In general, the majority of participants had a low level of knowledge (109, 62.6%). Gender was significantly associated with both knowledge (p = 0.021) and awareness (p = 0.039), as well as nationality (knowledge (p = 0.012) and awareness (p = 0.039)), and specialty training, which was significantly associated with both knowledge (p = 0.000) and awareness (p = 0.002).

Conclusion: Although all participants acknowledged that OSA is a clinical disease, their perspectives on screening and levels of confidence in utilizing screening techniques varied. While the participants exhibited commendable understanding in the majority of domains, there were some facets of OSA in which they lacked expertise.

## Introduction

Sleep apnea and hypopnea are common chronic conditions in the general population, and they are characterized by repeated episodes of apnea and hypopnea during sleep. It can manifest as obstructive sleep apnea (OSA), central sleep apnea (CSA), or a combination. When OSA disorder is combined with daytime sleepiness, the condition is known as obstructive sleep apnea-hypopnea syndrome [1, 2].

Obstructive sleep apnea is a common sleep disorder among middle-aged adults. It is defined as a sleep-related disorder in which airflow is significantly reduced due to complete or partial upper airway obstruction, resulting in oxygen deficiency and disrupted sleep [3, 4], and it is characterized by nocturnal symptoms such as snoring and witnessed apneas. Sleep disruption can lead to impairment of emotional, marital, social, and occupational health and increase accident risk because of excessive daytime sleepiness [4-6]. There are a lot of risk factors associated with OSA, such as obesity, male sex, older age, adenotonsillar hypertrophy, and smoking, which are established risk factors for OSA, with the additional risk associated with race/ethnicity and family history. The risk of OSA correlates with body mass index, and obesity remains the one major modifiable risk factor for OSA [7, 8].

Obstructive sleep apnea is a growing public health issue, particularly in developing countries, as it affects 3% to 7% of men and 2% to 5% of women [9-11] and may be associated with serious complications such as systemic hypertension, pulmonary hypertension, coronary artery diseases, heart failure, arrhythmias, stroke, and neurocognitive disorders [11, 12]. Obstructive sleep apnea has recently been identified as one of the most common undiagnosed chronic diseases, accounting for more mortality and morbidity than any other sleep disorder [11, 13]. It is important to recognize that untreated OSA has an independent impact on morbidity and mortality [14].

Primary care physicians can help ensure that OSA is properly diagnosed and treated by becoming more aware of the disorder and its impact on their patients' general health. Patients who are obese or snore should be suspected of having OSA [14].

This study aims to assess general practitioners' knowledge, awareness, and attitude regarding OSA in primary care in the Al-Baha region of Saudi Arabia, and the findings will help to write recommendations to primary care physicians regarding OSA.

## Materials and methods

Study design

A cross-sectional observational study design was employed from January 18, 2023, to August 18, 2023, focusing on primary healthcare physicians practicing in the Al-Baha region of Saudi Arabia.

Sample size calculation

The required sample size was determined using the Yamane formula, considering a confidence level of 95% and a margin of error of 7%. Adjusting for a 10% non-response rate, the final sample size was determined to be 161 primary healthcare physicians.

Yamane formula: (n = N/ 1+ N(e)2)

where n = number of samples; N = total population; e = error tolerance

Sampling method

A convenience sampling method was utilized to select participants from the sample frame of primary healthcare physicians practicing in the Al-Baha region.

Inclusion and exclusion criteria

Primary healthcare physicians working in the Al-Baha region were included in the study. Those who were not primary healthcare physicians or worked in secondary or tertiary hospitals, those with incomplete questionnaires, and those who refused to participate were excluded.

Data collection

Data were collected through phone interviews with primary healthcare physicians. The Obstructive Sleep Apnea Knowledge and Attitude (OSAKA) questionnaire was utilized. The questionnaire consists of three sections: a knowledge section with 18 questions scored from 0 to 18, an attitude section with five items significantly correlated with each other, and a section focusing on sociodemographic factors such as age, gender, specialty, subspecialty (if applicable), seniority, and workplace. Further, a prepared questionnaire to assess awareness was used, consisting of 18 questions with true, false, and I do not know options. Knowledge and awareness scores were further classified into low (<60% of the overall score), average (60% to 79% of the overall score), and high (>80% of the overall score). This category was included according to Bloom’s criteria and has been used by several studies in the past [15, 16].

Ethical considerations

Verbal consent was obtained from each participant at the beginning of the questionnaire. Participants were informed about the aims, methods, and anticipated benefits of the study. The study is self-financed, and the researchers are affiliated with the Faculty of Medicine at Al-Baha University, Al-Baha, Saudi Arabia. Potential risks are deemed minimal. Participants had the right to withdraw without reprisals, and they were offered a copy of the informed consent. The study did not impose any financial burden related to investigations or treatment. This research study was approved by the Committee of Research Ethics, Deanship of Scientific Research at Al Baha University, Kingdom of Saudi Arabia (approval number: REC/SUR//BU-FM/2023/05).

Data analysis

Data were entered into Microsoft Office Excel 2023 (Microsoft Corp., Redmond, WA) and analyzed using IBM Statistical Package for the Social Sciences (SPSS) software version 26 (IBM Corp., Armonk, NY). A descriptive analysis was performed to report sociodemographic information. A contingency table analysis (chi-square test) was conducted to compare the rates of unmatched samples. Independent t-tests were used for analyzing continuous variables.

## Results

Table 1 presents the demographic factors of the participants in the study, with a total sample size of 174 primary healthcare physicians.

**Table 1:** Demographic factors of the participants (N=174)

		Frequency (n=174)	Percentage
Gender	Male	101	58.0%
	Female	73	42.0%
Age (years)	Mean (SD)	32.73 (9.14)	
Nationality	Saudi	81	46.6%
	Non-Saudi	93	53.4%
Specialty training	Intern	15	8.6%
	General practitioner	103	59.2%
	Family physician	30	17.2%
	Internal medicine	12	6.9%
	Pediatrics	11	6.3%
	Obstetrician/Gynecologist	2	1.1%
	Otolaryngologist	1	0.6%
Years of experience	Less than 10 years	131	75.3%
	10-20 years	31	17.8%
	20-30 years	8	4.6%
	> 30 years	4	2.3%
Current job title	Resident	154	88.5%
	Specialist	14	8.0%
	Consultant	6	3.4%

The majority of participants were male (101, 58.0%), while 73 (42.0%) were female. The mean age of the participants was 32.73 years (SD = 9.14). Regarding nationality, 81 (46.6%) participants were Saudi, and 93 (53.4%) were non-Saudi. Among the participants, the most common specialty training was as a general practice (103, 59.2%), followed by family medicine(30, 17.2%), and internal medicine (12, 6.9%). The majority of participants had an experience duration of less than 10 years (131, 75.3%), while 31 (17.8%) had 10-20 years of experience. In terms of the current job title, most participants were residents (154, 88.5%), followed by specialists (14, 8.0%), and consultants six, 3.4%).

In Table 2, the knowledge of the participants regarding OSA using the OSAKA questionnaire and the correct answer rates for each item are reported.

**Table 2:** Knowledge of the participants toward obstructive sleep apnea using the OSAKA questionnaire. (N=174) OSAKA: Obstructive Sleep Apnea Knowledge and Attitude

	Correct answer	
	Frequency (n=174)	Percentage
Women with obstructive sleep apnea may present with fatigue alone.	103	59.2%
Uvulopalatopharyngoplasty is curative for the majority of patients with obstructive sleep apnea.	28	16.1%
The estimated prevalence of obstructive sleep apnea among adults is between 2% and 10%.	92	52.9%
The majority of patients with obstructive sleep apnea snore.	150	86.2%
Obstructive sleep apnea is associated with hypertension.	70	40.2%
An overnight sleep study is the gold standard for diagnosing obstructive sleep apnea	136	78.2%
Continuous positive airway pressure (CPAP) therapy may cause nasal congestion.	83	47.7%
Laser-assisted uvuloplasty is an appropriate treatment for severe obstructive sleep apnea.	17	9.8%
The loss of upper airway muscle tone during sleep contributes to obstructive sleep apnea.	128	73.6%
The most common cause of obstructive sleep apnea in children is the presence of large tonsils and adenoids.	150	86.2%
A craniofacial and oropharyngeal examination is useful in the assessment of patients with suspected obstructive sleep apnea.	125	71.8%
Alcohol at bedtime improves obstructive sleep apnea.	115	66.1%
Untreated obstructive sleep apnea is associated with a higher incidence of automobile crashes.	106	60.9%
In men, a collar size of 17 inches or greater is associated with obstructive sleep apnea.	61	35.1%
Obstructive sleep apnea is more common in women than in men.	60	34.5%
Continuous positive airway pressure is the first line of therapy for severe obstructive sleep apnea.	120	69.0%
Less than five apneas or hypopneas per hour is normal in adults.	43	24.7%
Cardiac arrhythmias may be associated with untreated obstructive sleep apnea.	111	63.8%

The item with the highest correct answer rate was "The majority of patients with obstructive sleep apnea snore" (150, 86.2%). This indicates that the participants had good knowledge of the association between snoring and OSA. On the other hand, the item with the lowest correct answer rate was "Laser-assisted uvuloplasty is an appropriate treatment for severe obstructive sleep apnea" (17, 9.8%). This suggests a lack of awareness among the participants regarding the appropriate treatment for severe OSA. Furthermore, it is noteworthy that the participants demonstrated relatively good knowledge regarding symptoms, diagnosis, and some aspects of the treatment of OSA. They correctly identified that OSA is associated with fatigue (103, 59.2%), hypertension (70, 40.2%), and cardiac arrhythmias (111, 63.8%). They also recognized the importance of overnight sleep studies for diagnosis (136, 78.2%) and continuous positive airway pressure (CPAP) therapy for nasal congestion (83, 47.7%). However, there were knowledge gaps in understanding the prevalence of OSA (92, or 52.9%) and the appropriate treatment options for severe OSA, such as uvulopalatopharyngoplasty (28, 16.1%) and laser-assisted uvulopalatoplasty (17, 9.8%).

**Figure 1:**
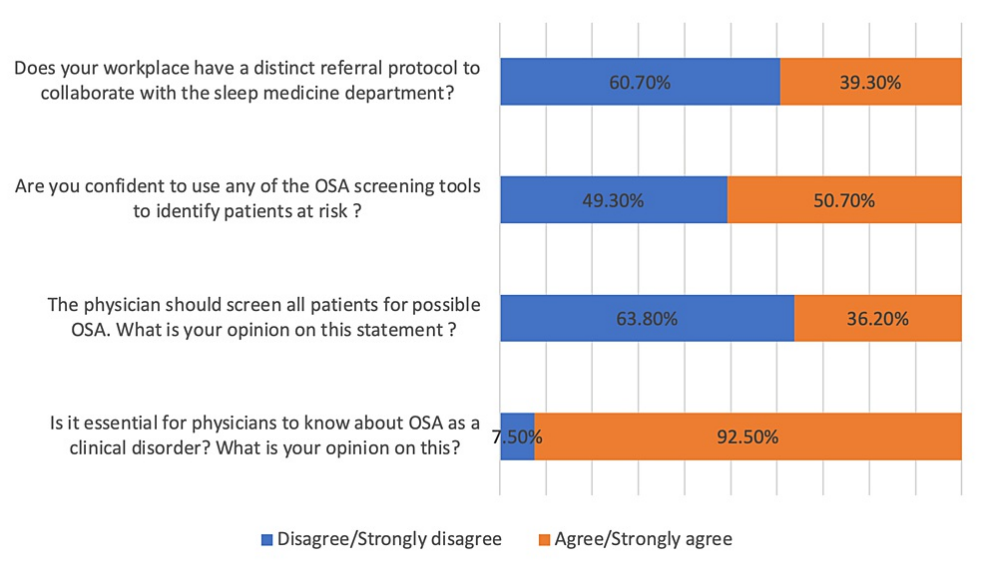
Attitude of the participants toward OSA (N=174) OSA: obstructive sleep apnea

Figure 1 provides insights into the attitudes of the participants toward OSA.

The majority of participants agreed that it is essential for physicians to know about OSA as a clinical disorder (161, 92.5%). However, when it came to screening all patients for possible OSA, there was a higher proportion of participants who disagreed or strongly disagreed (111, 63.8%) compared to those who agreed or strongly agreed (63, 36.2%). Additionally, there was a relatively equal distribution of opinions regarding confidence in using OSA screening tools, with 85 (49.3%) agreeing and 89 (50.7%) disagreeing. Regarding the presence of a distinct referral protocol in their workplace to collaborate with the sleep medicine department, there was a higher proportion of participants who disagreed or strongly disagreed (105, 60.7%) compared to those who agreed or strongly agreed (69, 39.3%).

In Table 3, the participants' level of awareness regarding various aspects of OSA is presented.

**Table 3:** Awareness of the participants regarding OSA (N=174) OSA: obstructive sleep apnea

	Correct answer	
	Frequency (n=174)	Percentage
In Saudi Arabia, the overall prevalence of OSA, as defined by the American Academy of Sleep Medicine, was 8.8%.	65	37.4%
OSA is described as frequent episodes of partial or complete obstruction of the upper airway for at least 60 seconds with accompanied respiratory efforts.	132	75.9%
OSA is more common in women than in men.	66	37.9%
OSA leads to sleep fragmentation and hyperoxia.	101	58.0%
Bed partners usually complain of loud snoring and/or breathing interruptions during sleep (observed apnea).	145	83.3%
Type 2 diabetes mellitus may be linked to OSA.	84	48.3%
The gold standard for OSA diagnosis is polysomnography.	101	58.0%
Some OSA patients complain of fatigue, daytime sleepiness, morning headaches, and/or waking with breath-holding, choking, or gasping.	153	87.9%
OSA is more common in people with a low body mass index.	122	70.1%
Physicians are responsible for OSA cases diagnosis.	139	79.9%
OSA is common in people with a neck circumference of less than 18 inches.	62	35.6%
Some orofacial pain and bruxism are linked to OSA.	73	42.0%
The physician's role is to refer potential patients who are at high risk of OSA to a sleep physician.	129	74.1%
Untreated or undiagnosed OSA could predispose patients to systemic hypertension.	110	63.2%
Untreated OSA is associated with an increased risk of mortality (heart attack or stroke).	133	76.4%
OSA is seen more in people younger than 40.	75	43.1%
Continuous positive airway pressure (CPAP) is the first line of treatment for mild, moderate, and severe obstructive sleep apnea.	115	66.1%
Untreated OSA puts patients at a high risk of automobile accidents.	133	76.4%

The correct answer rates for each item are reported. Overall, the participants demonstrated good awareness in most areas, with correct answer rates ranging from 62 (35.6%) to 153 (87.9%). However, there were certain items where awareness was relatively lower, such as the prevalence of OSA in Saudi Arabia (65, 37.4%), the fact that OSA is more common in women than men (66, 37.9%), and the association of orofacial pain and bruxism with OSA (73, 42.0%).

**Figure 2:**
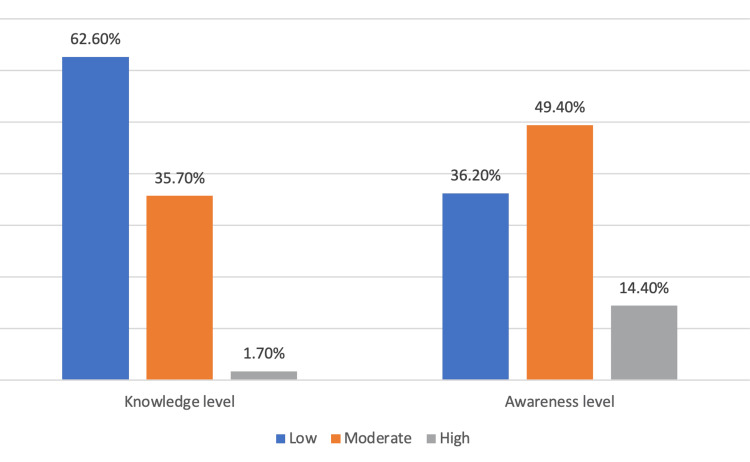
The level of knowledge and awareness of OSA (N=174) OSA: obstructive sleep apnea

Figure 2 illustrates the level of knowledge and awareness of the participants toward OSA.

In terms of knowledge, the majority of participants had a low level of knowledge (109, 62.6%), followed by a moderate level (62, 35.6%), and a small proportion had a high level of knowledge (3, 1.7%). Regarding awareness, a similar pattern was observed, with 63 (36.2%) having a low level of awareness, 86 (49.4%) having a moderate level of awareness, and 25 (14.4%) having a high level of awareness.

Table 4 examines the relationship between demographic factors and the participants' knowledge and awareness of OSA.

**Table 4:** The relationship between demographic factors and knowledge and awareness of obstructive sleep apnea (N = 174) Significant p-value (p <0.001)

		Knowledge categories							Awareness						
		Low		Moderate		High			Low		Moderate		High		
		Count	Row N %	Count	Row N %	Count	Row N %	p-value	Count	Row N %	Count	Row N %	Count	Row N %	p-value
Gender	Male	67	66.3%	34	33.7%	0	0.0%	0.085	43	42.6%	49	48.5%	9	8.9%	0.021*
	Female	42	57.5%	28	38.4%	3	4.1%		20	27.4%	37	50.7%	16	21.9%	
Nationality	Saudi	57	70.4%	21	25.9%	3	3.7%	0.012*	36	44.4%	38	46.9%	7	8.6%	0.039*
	Non-Saudi	52	55.9%	41	44.1%	0	0.0%		27	29.0%	48	51.6%	18	19.4%	
Specialty training	Intern	12	80.0%	3	20.0%	0	0.0%	0.000*	9	60.0%	6	40.0%	0	0.0%	0.002*
	General practitioner	64	62.1%	39	37.9%	0	0.0%		36	35.0%	58	56.3%	9	8.7%	
	Family physician	15	50.0%	15	50.0%	0	0.0%		13	43.3%	10	33.3%	7	23.3%	
	Internal medicine	8	66.7%	4	33.3%	0	0.0%		3	25.0%	5	41.7%	4	33.3%	
	Pediatrics	8	72.7%	0	0.0%	3	27.3%		2	18.2%	6	54.5%	3	27.3%	
	Obstetrician/Gynecologist	2	100.0%	0	0.0%	0	0.0%		0	0.0%	0	0.0%	2	100.0%	
	Otolaryngologist	0	0.0%	1	100.0%	0	0.0%		0	0.0%	1	100.0%	0	0.0%	
Years of experience	Less than 10 years	86	65.6%	42	32.1%	3	2.3%	0.249	45	34.4%	70	53.4%	16	12.2%	0.011*
	10-20 years	15	48.4%	16	51.6%	0	0.0%		12	38.7%	14	45.2%	5	16.1%	
	20-30 years	4	50.0%	4	50.0%	0	0.0%		2	25.0%	2	25.0%	4	50.0%	
	>30 years	4	100.0%	0	0.0%	0	0.0%		4	100.0%	0	0.0%	0	0.0%	
Current job title	Resident	100	64.9%	51	33.1%	3	1.9%	0.226	58	37.7%	79	51.3%	17	11.0%	0.004*
	Specialist	5	35.7%	9	64.3%	0	0.0%		5	35.7%	3	21.4%	6	42.9%	
	Consultant	4	66.7%	2	33.3%	0	0.0%		0	0.0%	4	66.7%	2	33.3%	
Is it essential for physicians to know about OSA as a clinical disorder? What is your opinion on this?	Disagree/strongly disagree	9	69.2%	4	30.8%	0	0.0%	0.805	7	53.8%	4	30.8%	2	15.4%	0.332
	Agree/strongly agree	100	62.1%	58	36.0%	3	1.9%		56	34.8%	82	50.9%	23	14.3%	

Several demographic factors showed significant associations with knowledge and awareness. Gender was significantly associated with both knowledge (p = 0.021) and awareness (p = 0.039), with a higher proportion of females having moderate knowledge and awareness levels compared to males. Nationality also showed a significant association with both knowledge (p = 0.012) and awareness (p = 0.039), with a higher proportion of non-Saudi participants having moderate knowledge and awareness levels. Specialty training was significantly associated with both knowledge (p = 0.000) and awareness (p = 0.002), with participants specializing in internal medicine, pediatrics, and otolaryngology demonstrating higher knowledge and awareness levels. Years of experience showed a significant association with awareness (p = 0.011), with participants having less than 10 years of experience showing higher awareness levels. The current job title was significantly associated with both knowledge (p = 0.004) and awareness (p = 0.004), with specialists and consultants demonstrating higher knowledge levels, while residents showed higher awareness levels.

## Discussion

Obstructive sleep apnea is a medical condition of considerable clinical importance, necessitating the involvement of primary care physicians and other healthcare practitioners [17]. The purpose of this research was to determine the knowledge, awareness, and attitudes of primary care physicians in Saudi Arabia regarding OSA. Our findings provide insights into the participants' attitudes, level of knowledge, and awareness regarding OSA, as well as the relationship between these variables and a variety of demographic parameters.

A significant proportion of the participants in our research acknowledged the criticality of physicians possessing an understanding of OSA as a clinical condition. This is consistent with prior studies that emphasize the importance of OSA to the well-being and overall livability of individuals [18]. However, regarding the screening of all patients for potential OSA, a significant majority of respondents were opposed or extremely opposed. The aforementioned finding gives rise to apprehensions over the inadequate diagnosis and treatment of OSA, given the critical nature of early detection in facilitating successful management [19]. This implies that additional awareness campaigns and educational initiatives may be required to underline the significance of OSA screening in primary care settings.

Numerous existing texts suggest that self-confidence is an essential feature of a great physician, including primary care physicians [20, 21]. In terms of their confidence in utilizing OSA screening techniques, the participants' viewpoints were comparatively balanced. This result may be attributable to the absence of universally acknowledged and standardized screening instruments for OSA in primary care settings [22]. Similar to this study’s findings, another study by Chang et al. compared primary care physicians' attitudes toward OSA in three African regions, and they reported that their study participants had low confidence in caring for OSA patients [23]. Hence, it is imperative to prioritize the development and validation of dependable and user-friendly screening instruments that can be efficiently integrated into primary care environments.

Concerning awareness and understanding, our research unveiled a spectrum of accurate response rates for different facets of OSA. Although the participants generally exhibited a high level of awareness, there were specific items for which their level of awareness was comparatively lower. An example of the participants' poor knowledge pertains to the prevalence of OSA in Saudi Arabia, the gender-based disparity in OSA prevalence between men and women, and the correlation between OSA and bruxism and orofacial pain. The aforementioned knowledge gaps align with prior research that has documented inadequate levels of awareness among healthcare workers concerning diverse facets of OSA [15]. In line with the research conducted by Embarak et al. [17] and Corso et al. [16], our study's findings revealed a substantial range of variation in the knowledge questionnaire items, from 35.6% to 87.9%. This result indicates that understanding OSA is insufficient and requires curriculum-appropriate instruction. According to our research, the item about snoring received the highest proportion of accurate responses from primary care physicians. This discovery is consistent with the results of a study conducted in Egypt in 2020 [17]. The majority of participants in this survey (150, 86.2%), concurred that the majority of patients with OSA snore.

The participants exhibited a low to moderate degree of overall knowledge and awareness, with just a minority displaying a high level of knowledge and awareness. Similar results were reported by recent studies conducted in Saudi Arabia and Ecuador, which revealed that the mean score of the respondents was approximately 10 out of 18 [22, 24]. On the contrary, a few alternative studies have documented somewhat higher average knowledge scores among the participants [16, 25]. This finding underscores the necessity for focused educational efforts aimed at enhancing primary healthcare practitioners' comprehension of OSA. Prior research has demonstrated that educational initiatives and interventions might efficaciously augment the understanding and consciousness of OSA in healthcare [26]. Hence, the integration of OSA education into medical school curricula and the provision of ongoing professional development opportunities for practicing physicians may contribute to the resolution of the knowledge deficit.

Our study offers useful insights into the correlation between demographic characteristics and knowledge and awareness of OSA. The variables of gender, nationality, current job title, specialist training, and experience duration showed strong correlations with levels of knowledge and awareness. Higher levels of knowledge and awareness were observed among females, non-Saudi participants, those who specialized in internal medicine, pediatrics, and otolaryngology, persons with fewer than 10 years of experience, and specialists and consultants. The results of this study align with prior research that has demonstrated discrepancies in OSA knowledge and awareness according to demographic attributes [22, 27]. The authors emphasize the need to take into account these variables during the development of training interventions and customize them to suit the particular requirements of distinct healthcare professional cohorts.

Several limitations of our research should be duly recognized. To begin with, it should be noted that the sample size was very small, which raises concerns over the representativeness of primary healthcare providers in Saudi Arabia as a whole. Furthermore, the research was executed exclusively within a certain geographic area, thereby constraining the applicability of the results. Furthermore, self-reported data, which are susceptible to recall and social desirability biases, were utilized in the study. Further investigation utilizing more extensive sample sizes and a broader range of participants is imperative to authenticate and broaden the scope of our findings.

## Conclusions

In conclusion, although all participants acknowledged that OSA is a clinical disease, their perspectives on screening and levels of confidence in utilizing screening techniques varied. While the participants exhibited commendable understanding in the majority of domains, there were some facets of OSA in which they lacked expertise. The prevailing levels of knowledge and awareness were primarily moderate to low, underscoring the criticality of implementing focused educational interventions. A correlation was seen between demographic variables and levels of knowledge and awareness, underscoring the criticality of customizing educational interventions to suit certain cohorts of healthcare professionals. By enhancing the expertise and consciousness of primary care physicians, it is possible to improve the detection and treatment of OSA, thereby leading to enhanced patient outcomes and quality of life.
